# Differential gene expression in Glisson’s capsule associated with liver abscesses in feedlot cattle

**DOI:** 10.1093/jas/skag320

**Published:** 2026-10-06

**Authors:** Macy T Lawrence, Trent E Schwartz, Loni W Lucherk, Ty E Lawrence, Emilie C Baker

**Affiliations:** Department of Agricultural Sciences, West Texas A&M University, Canyon, TX 79016, United States; Department of Agricultural Sciences, West Texas A&M University, Canyon, TX 79016, United States; Department of Agricultural Sciences, West Texas A&M University, Canyon, TX 79016, United States; Department of Agricultural Sciences, West Texas A&M University, Canyon, TX 79016, United States; Department of Agricultural Sciences, West Texas A&M University, Canyon, TX 79016, United States

**Keywords:** animal welfare, liver abscesses, inflammation, nociception, pain signaling, tissue remodeling

## Abstract

Liver abscesses (LA) in feedlot cattle are associated with reduced growth performance, increased liver and offal condemnations, and carcass value losses; however, potential animal welfare impacts, including pain, remain poorly understood. Although liver parenchyma lacks pain receptors, stretching or inflammation of the surrounding Glisson’s capsule is known to cause pain in humans. As the liver swells in response to LA, expansion of Glisson’s capsule may activate nociceptive pathways. Our objective was to compare gene expression in Glisson’s capsule from livers with (*n* = 6) and without LA (*n* = 9) to identify molecular signatures associated with LA. RNA was extracted from Glisson’s capsule tissue, sequenced on an Illumina platform, aligned to the Bos taurus reference genome using HISAT2, and quantified with featureCounts. Differential gene expressions were assessed using DESeq2 (FDR ≤ 0.05; |log2 FC| ≥ 1). Sixty genes were differentially expressed; 41 were upregulated and 19 were downregulated in ABS tissue. *FOS* (+1.6 log2FC) and *FOSB* (+2.4 log2FC), molecular markers associated with acute and chronic pain, respectively, were upregulated in animals with LA. ENSBTAG00000061094, the bovine ortholog of human *MRP4*, had the greatest differential expression (−4.5 log2FC) and was downregulated in animals with LA; *MRP4* is involved in inflammatory mediator transport and altered inflammatory pain thresholds. *SNCA* (−2.1 log2FC) has been associated with pain hypersensitivity and was downregulated in animals with LA, whereas *ISG12(B)* (+3.3 log2FC) indicated heightened innate immune and mitochondrial apoptotic activity and was upregulated in animals with LA. Increased *EPHB3* (+1.8 log2FC) was upregulated in animals with LA and is consistent with signaling implicated in inflammatory remodeling and peripheral neuroimmune pain pathways. This study provides the first characterization of gene expression changes in Glisson’s capsule associated with bovine LA. Differentially expressed genes related to inflammation, nociceptive signaling, and tissue stress suggest that LA creates an inflamed and likely pain-relevant molecular environment within the liver capsule. Further research is needed to confirm whether these molecular changes correspond to perceived pain in cattle.

## Teaser

Liver abscesses in feedlot cattle were associated with changes in Glisson’s capsule gene expression related to inflammation, tissue stress, and pain-signaling pathways. These findings provide new insight into the likely welfare implications of liver abscesses and highlight the need for further research to confirm whether these molecular changes correspond to perceived pain in cattle.

## Introduction

Liver abscesses (LA) are a common condition in feedlot cattle, most commonly associated with high-concentrate diets, and range in prevalence from 0% to 95.5% of cattle within a lot at slaughter (Grimes et al. 2024). LA have been associated with increased liver and offal condemnation rates (Herrick et al. 2022), reduced carcass performance (Brown and Lawrence 2010; Grimes et al. 2024), and increased trimming losses throughout the slaughter process (Montgomery 1985), which culminate in substantial economic losses (Herrick et al. 2022; Broadway et al. 2024; Grimes et al. 2024).

In human medicine, LA are known to cause inflammation of the liver parenchyma and are well documented to cause pain (Krige and Beckingham 2001; Kozielewicz et al. 2021; Leerapun et al. 2021). Historically, viscera tissue was believed to be insensitive to pain; however, subsequent research has demonstrated that pain can arise from a multitude of bodily mechanisms, including stretching of the connective tissue surrounding visceral organs, which can include the liver, spleen, and pancreas (Procacci et al. 1986). Glisson’s capsule is a connective tissue layer surrounding the parenchyma of the liver and protects liver tissue from trauma. The capsule forms a relatively thick connective tissue sheathing layer on the surface that is continuous with the intrahepatic portal sheath and is highly innervated by sensory nerve fibers and nociceptors. Visceral pain from the liver occurs almost exclusively when a lesion, such as abscess, stretches, distends, or causes inflammatory swelling against the capsule. Inflammation of the liver capsule has been linked to hepatic pain in humans (Reed 1884; Kaga et al. 2023). Right shoulder pain has also been associated with Glisson’s capsule injury (Ali et al. 2024; Dhamala et al. 2026). Furthermore, clinical studies have reported LA can cause moderate pain in humans, reported as a 6 out of 10 on a subjective human 10-point pain scale (Yang et al. 2025). Pain is a complex physiological process that emerges from interactions among inflammatory signaling pathways, nociceptive sensory pathways (Gold and Gebhart 2010; Pinho-Ribeiro et al. 2017), and modulatory neuroimmune mechanisms. Previous researchers (Basak et al. 2017) have additionally studied cauterization of the Glisson’s capsule in human medicine (typically during a cholecystectomy) and have reported postoperative liver pain associated with this procedure. Single-cell transcriptomics have been used in chronic pain research in humans (Kupari et al. 2021), however, there are no FDA-approved transcriptomic biomarkers for pain. Genes have different functions within differing tissue types, and pinpointing tissues that are relevant to pain-signaling is important for biomarker studies.

Limited studies have evaluated perceived pain associated with LA presence in animals. Textbook descriptions of clinical signs of a LA include abdominal pain, indicated by an arched back in cattle (Nagaraja and Chengappa 1998; Scott 2013). Signs of pain are additionally listed as a symptom when LA has been found in dogs (Farrar et al. 1996; Schwarz et al. 1998) and cats (Sergeeff et al. 2004).

Although causes for bovine LA and methods to reduce bovine LA have been well studied, potential animal welfare impacts, including pain, remain poorly understood and largely unexplored. Human studies indicate that Glisson’s capsule serves as a site of pain and nociceptive signaling. We hypothesized that nociceptive stimuli are generated within bovine liver capsule tissue as a result of liver deformation caused by the presence of LA. Our objective was to characterize differential gene expression in Glisson’s capsule between abscessed and non-abscessed livers.

## Materials and methods

Institutional Animal Care and Use Committee approval was not requested because no live animals were used in this experiment.

### Sample collection

Livers with and without LA were identified by visual inspection immediately after evisceration in a beef processing facility. All animals were less than 30 months of age and originated from 23 different feedlots in the Texas Panhandle region. A section of the liver surface (*n* = 23; abscessed = 7, healthy = 16) was excised, and the capsular layer was carefully peeled away from the underlying parenchyma (Figure 1). For abscessed livers, capsular tissue was removed from the exterior surface of the abscess and the area directly adjacent to the abscess. Seven of 23 animals (31.8%) exhibited LA, with 6 of the 7 abscesses classified as severe (A+). Capsule samples were rinsed in ice-cold phosphate-buffered saline, immediately placed on dry ice, and gently scraped to remove any residual hepatic tissue. Because Glisson’s capsule is thin (< 1 mm) and attached to the hepatic parenchyma, the excised tissue represents the capsule and its immediate microenvironment rather than a histologically purified cell population, and transcriptomic signals should be interpreted as reflecting this broader capsular environment. Capsule samples were stored at −80 °C, and shipped to Novogene (Sacramento, CA, USA) on dry ice for RNA extraction.

**Figure 1:**
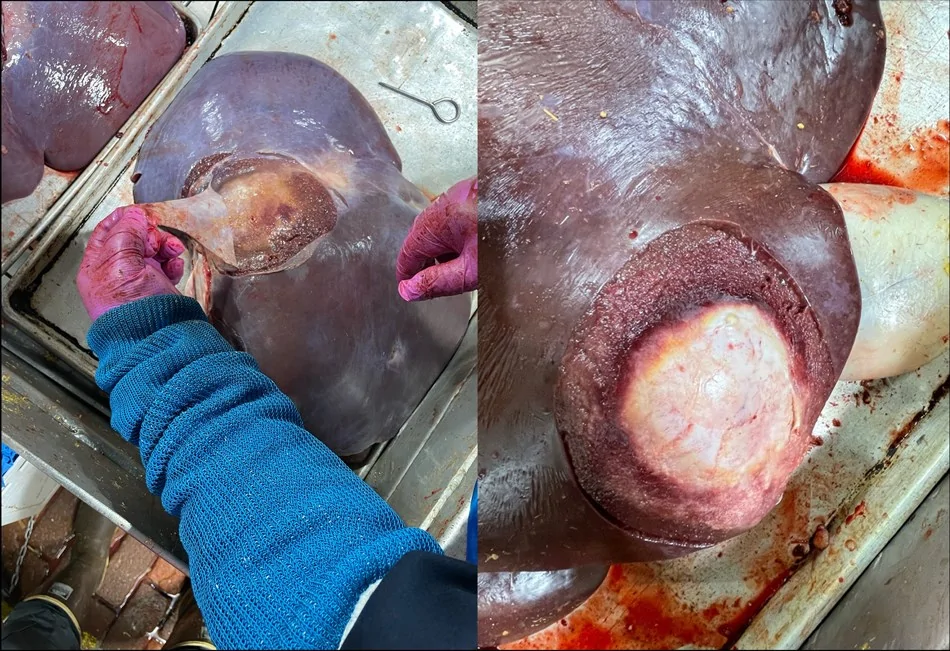
Visual representation of Glisson’s capsule removal from the area surrounding a liver abscess.

### Total RNA isolation and sequencing

Total RNA was isolated by homogenizing frozen tissue in TRIzol reagent using an automated tissue homogenizer, followed by chloroform phase separation, isopropanol precipitation, and ethanol washing. RNA concentration and purity were assessed by NanoDrop spectrophotometry, and RNA integrity number (RIN) was determined using an Agilent TapeStation system prior to library preparation.

Poly(A)-enriched mRNA libraries were prepared using the Plant and Animal Eukaryotic mRNA (WOBI) pipeline (Novogene, Sacramento, CA, USA) and sequenced on an Illumina NovaSeq X Plus platform to generate paired-end 150 bp reads, targeting 30 million read pairs per sample. Adapter trimming specifically targeted the Illumina P5 and P7 adapter sequences flanking the inserts, including their associated i5 and i7 index regions, to remove any residual adapter-derived bases from read ends before downstream analysis. After adapter removal, reads with more than 10% undetermined bases or with over 50% of bases having a quality score ≤ 5 were discarded, and only these cleaned paired-end reads were used for gene expression profiling. Reads were aligned to the ARS UCD1.3 bovine reference genome using HISAT2 (Kim et al. 2019, v2.2.1) with default parameters for paired-end data. Transcript integrity numbers (TIN) were calculated from aligned BAM files using RSeQC (v5.0.4, Wang et al. 2012). Aligned reads were summarized at the gene level with featureCounts (Liao et al. 2014, Subread, 2.1.1) using Ensembl bovine gene annotations (ARS UCD1.3).

### Bioinformatic analysis

Differential gene expression analysis was performed in R using the DESeq2 (Love et al. 2014) package (v 1.46.0), starting from the untransformed gene level count matrix. A DESeqDataSet was constructed from the count matrix with abscess status (abscessed vs. non-abscessed) specified as the explanatory variable (design = ∼ condition). All samples were processed concurrently, minimizing batch-related technical variation. Feedlot of origin was not included as a covariate, as each of the animals originated from a different feedlot, making this variable non-identifiable as a term independent of individual animal identity. To reduce the influence of lowly expressed genes, transcripts were filtered prior to differential expression analysis by retaining only genes with a minimum count of 10 reads in at least as many samples as the smallest experimental group. Genes failing to meet this threshold were excluded from downstream analyses. DESeq2 models the remaining counts with a negative binomial distribution, estimates size factors to normalize for library depth, and then estimates and shrinks gene-wise dispersion parameters before fitting the model. Wald tests were used to assess differential expression between LA and non-abscess groups, and *P*-values were adjusted for multiple testing using the Benjamini–Hochberg false discovery rate procedure. Genes were considered differentially expressed if FDR ≤ 0.05 and ∣log2 FC∣≥1.

Functional enrichment analysis was performed on differentially expressed genes using g: Profiler (Kolberg et al. 2023), querying Gene Ontology biological process, molecular function, and cellular component terms, as well as KEGG, Reactome, and WikiPathways. Enrichment *P*-values were corrected for multiple testing using the g: Profiler g: SCS (set counts and sizes) method, and terms with adjusted *P*-values < 0.05 were considered statistically significant.

## Results

RIN for Glisson’s ranged from 4.1 to 8.5. RNA integrity values did not differ significantly between abscessed and healthy groups (Wilcoxon rank-sum test, *P *= 0. 9701) indicating that RNA quality was not confounded with abscess status prior to differential expression analysis. Samples with a RIN < 6.0 were excluded from further analysis, leaving 15 samples (abscessed = 6, healthy = 9; mean RIN 7.0; range 6.0–8.5). Of the eight excluded samples, one was from the abscessed group, and seven were from the healthy group; this exclusion was not significantly associated with abscess status (Fisher’s exact test, *P *= 0.345), indicating that sample loss due to RIN-based quality filtering was not disproportionately concentrated in either group. TIN were calculated for each library (median TIN = 73.50; range 68.06–77.16); no outliers were detected (Tukey’s method; values beyond 1.5× IQR from Q1/Q3). The median TIN did not differ (Wilcoxon rank-sum test*, P *= 0.9546) between abscessed and healthy groups. The final dataset comprised six Glisson’s capsule samples from abscessed livers (ABS) and nine Glisson’s capsules from non-abscessed livers (healthy) for differential expression analysis.

Descriptive comparisons were made between the six cattle with severe LA and the non-abscessed group. Mean live weight was similar between cattle with severe LA and non-abscessed cattle (730.7 ± 34.4 kg vs. 730.3 ± 76.2 kg, respectively). Likewise, mean hot carcass weight differed little between groups (469.5 ± 22.3 kg vs. 477.6 ± 54.2 kg). However, cattle with severe LA had numerically lower marbling scores (Small^69^ ± 5.6 vs. Small^85^ ± 10.3) and numerically lower calculated yield grades (2.64 ± 0.51 vs. 3.29 ± 1.07) than non-abscessed cattle.

Alignment to the ARS UCD1.3 reference genome using HISAT2 yielded a mean overall alignment rate of 96.6% (range: 96.1%–96.9%) across the 15 samples retained for differential expression analysis. On average, 91.5% of read pairs (range: 90.2%–92.3%) aligned concordantly to a single unique locus, with an additional 2.8% (range: 2.4%–3.6%) aligning concordantly to multiple loci. Mean sequencing depth was approximately 37.1 million read pairs per sample (range: 30.4%–49.6 million), consistent with the targeted depth of 30 million read pairs.

After filtering out low or non-expressed transcripts, 17,626 genes remained for analysis. Principal component analysis of variance-stabilized counts (Figure 2) indicated only partial separation between ABS and healthy samples, with some overlap between groups. This overlap may reflect variability in abscess chronicity, as the duration of abscess presence prior to slaughter was not known and the capsular response likely evolves over the course of abscess development, potentially contributing to heterogeneity within the ABS group. Using thresholds of FDR ≤ 0.05 and ∣log2 FC∣≥1, 60 genes were identified as differentially expressed between ABS and healthy. Of these, 41 genes were upregulated in the ABS group relative to the healthy group (Table 1), whereas 19 genes were downregulated in the ABS samples (Table 2). Forty-one genes were upregulated with LA presence, including *C1QTNF6* (tumor necrosis factor related protein 6), *COL6A6* (collagen type VI alpha 6 chain), *FKBP7* (FKBP prolyl isomerase 7), *FOS* (Fos proto-oncogene, AP-1transcription factor subunit), *IFI27L2* (interferon alpha inducible protein 27 like 2), *IGSF10* (immunoglobulin superfamily member 10), *P4HA2* (prolyl 4-hydroxylase subunit alpha-2), and *PDGFRL* (platelet-derived growth factor receptor-like) (*P *< 0.01); *BOC* (brother of CDO), *C1QTNF3* (complement C1q tumor necrosis factor-related protein 3), *CPZ* (carboxypeptidase Z), *FOSB* (FosB proto-oncogene, AP-1 transcription factor subunit), *HSPA6* (heat shock protein family A member 6), and *RRAD* (Ras-related associated with diabetes) (*P *= 0.01); *CRTAC1* (cartilage acidic protein 1), *GULP1* (PTB domain-containing engulfment adaptor 1), *EPHB3* (EPH receptor B3), *NEGR1* (neuronal growth regulator 1), *SCG5* (secretogranin V), *SERF1B* (small EDRF-rich factor 1B), and *SYT1* (synaptotagmin 1) (*P *= 0.02); *CCDC160* (coiled-coil domain containing 160), *DOK6* (dedicator of cytokinesis 6), *GAL3ST4* (galactose-3-O-sulfotransferase 4), *MXRA8* (matrix remodeling associated 8), and *TMEM92* (transmembrane protein 92) (*P *= 0.03); *DNM1* (dynamin 1), *FNDC1* (fibronectin type III domain containing 1), *FZD2* (frizzled class receptor 2), *LIPG* (lipase G, endothelial type), and *LRP3* (low-density lipoprotein receptor-related protein 3) (*P *= 0.04); and *CD99* (single-chain type-1 glycoprotein), *EDIL3* (EGF-like repeat and discoidin I-like domain-containing protein 3), *LGALS1* (lectin, galactoside-binding, soluble, 1), *LOX* (lysyl oxidase), *NR4A2* (nuclear receptor subfamily 4 group A member 2), *RCN3* (reticulocalbin 3), and *SPARC* (secreted protein acidic and rich in cysteine) (*P *= 0.05). Nineteen genes were downregulated with LA presence, including *CDHR5* (cadherin-related family member 5), *MAT1A* (methionine adenosyltransferase 1A), *SNCA* (synuclein alpha), and *ZASP* (Z-band alternatively spliced PDZ-motif) (*P *< 0.01); *APCS* (amyloid P component, serum), *NID1* (nidogen 1), *SLFN12* (schlafen family member 12), and *TMED6* (transmembrane emp24 domain-containing protein 6) (*P *= 0.01); *INMT* (indolethylamine N-methyltransferase) (*P *= 0.02); *ABCC4* (ATP-binding cassette sub-family C member 4) and *TM7SF2* (transmembrane 7 superfamily member 2) (*P *= 0.03); and *BCL11A* (B-cell lymphoma/leukemia 11A) (*P *= 0.05). Functional enrichment of the upregulated genes yielded a single significant Gene Ontology cellular component term, “extracellular region,” and two WikiPathways, “Corticotropin releasing hormone” and “EGF EGFR signaling pathway” (Table 3). Functional enrichment of downregulated genes yielded a single Gene Ontology Molecular Function term, “calcium ion binding.”

**Figure 2:**
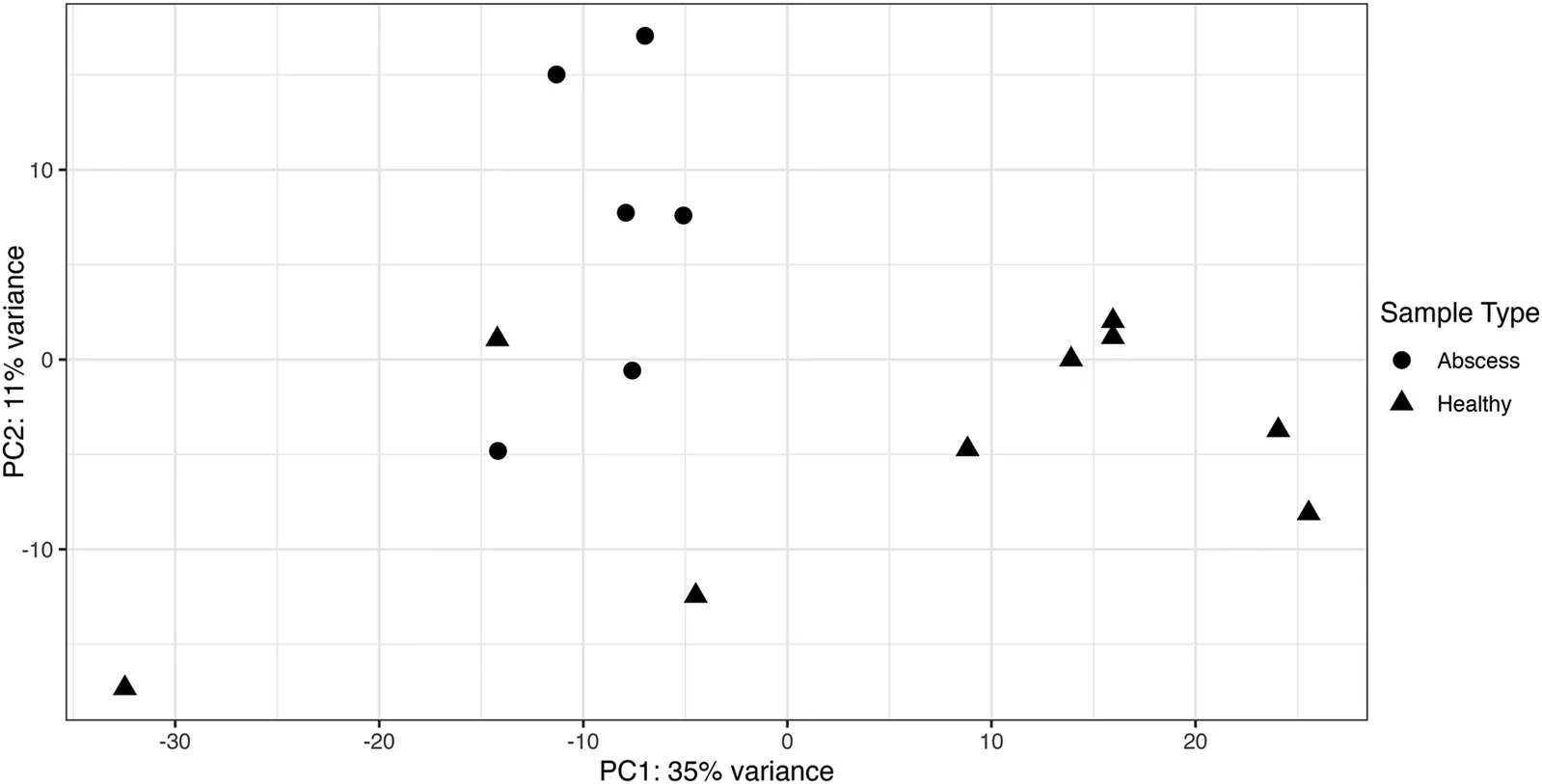
Principal component analysis of variance-stabilized gene expression counts.

**Table 1:** Upregulated differentially expressed genes within Glisson’s capsule for cattle with a liver abscess.

Name	Gene symbol	Gene name	Log2 -fold change^1^	False discovery rate *P-*value
**ENSBTAG00000004272**	IFI27L2	Interferon alpha-inducible protein 27 Like 2A	3.284	< 0.01
**ENSBTAG00000076424**	UBA1	Ubiquitin like modifier activating enzyme 1	2.559	< 0.01
**ENSBTAG00000008074**	C1QTNF6	Complement C1q tumor necrosis factor-related protein 6	2.136	< 0.01
**ENSBTAG00000001728**	IGSF10	Immunoglobulin superfamily member 10	1.922	< 0.01
**ENSBTAG00000012837**	COL6A6	Collagen type VI alpha 6 chain	1.743	< 0.01
**ENSBTAG00000004322**	FOS	c-Fos protein	1.550	< 0.01
**ENSBTAG00000001966**	PDGFRL	Platelet-derived growth factor receptor-like Protein	1.498	< 0.01
**ENSBTAG00000018281**	GPX7	Glutathione peroxidase 7	1.260	< 0.01
**ENSBTAG00000001116**	P4HA2	Prolyl-4-hydroxylase subunit alpha-2	1.242	< 0.01
**ENSBTAG00000001097**	FKBP7	Binding protein FK506	1.027	< 0.01
**ENSBTAG00000039035**	HSPA6	Heat shock protein family A Hsp70 member 6	2.467	0.01
**ENSBTAG00000008182**	FOSB	FosB Proto-oncogene AP-1Transcription factor subunit	2.420	0.01
**ENSBTAG00000017071**	C1QTNF3	Complement C1q tumor necrosis factor-related protein 3	2.149	0.01
**ENSBTAG00000013909**	BOC	BOC cell adhesion associated, oncogene regulated	1.572	0.01
**ENSBTAG00000014803**	CPZ	Carboxypeptidase Z	1.559	0.01
**ENSBTAG00000013929**	RRAD	Ras-related associated with diabetes	1.129	0.01
**ENSBTAG00000034693**	SYT1	Synaptotagmin-1	2.268	0.02
**ENSBTAG00000032613**	SCG5	Neuroendocrine protein 7B2	2.091	0.02
**ENSBTAG00000008102**	CRTAC1	Cartilage Acidic protein 1	2.064	0.02
**ENSBTAG00000005313**	EPHB3	Ephrin receptor B3	1.786	0.02
**ENSBTAG00000007652**	RFTN2	Raftlin-2	1.356	0.02
**ENSBTAG00000055278**	NEGR1	Neuronal growth regulator 1	1.331	0.02
**ENSBTAG00000007141**	GULP1	Engulfment adaptor PTB domain containing 1	1.314	0.02
**ENSBTAG00000034360**	SERF1B	Small EDRK-rich factor 1B	1.046	0.02
**ENSBTAG00000064591**	TMEM92	Transmembrane protein 92	2.412	0.03
**ENSBTAG00000036349**	CCDC160	Coiled-coil domain containing 160	1.620	0.03
**ENSBTAG00000046957**	DOK6	Docking protein 6	1.451	0.03
**ENSBTAG00000015017**	GAL3ST4	Galactose-3-O-sulfotransferase 4	1.134	0.03
**ENSBTAG00000012247**	MXRA8	Matrix remodeling associated 8	1.015	0.03
**ENSBTAG00000003938**	FNDC1	Fibronectin type III domain-containing protein 1	2.100	0.04
**ENSBTAG00000048916**	LIPG	Lipase endothelial	1.638	0.04
**ENSBTAG00000020590**	FZD2	Frizzled-2	1.552	0.04
**ENSBTAG00000047062**	LRP3	Low-density lipoprotein receptor-related protein 3	1.515	0.04
**ENSBTAG00000011307**	DNM1	Dynamin-1	1.361	0.04
**ENSBTAG00000044033**	EDIL3	EGF-like repeats and discoidin I-like domains 3	1.791	0.05
**ENSBTAG00000008114**	CD99	O-glycosylated transmembrane protein	1.684	0.05
**ENSBTAG00000003650**	NR4A2	Nuclear receptor subfamily 4 group A member 2	1.564	0.05
**ENSBTAG00000012994**	LOX	Lysyl oxidase	1.449	0.05
**ENSBTAG00000014835**	SPARC	Secreted protein acidic and rich in cysteine	1.357	0.05
**ENSBTAG00000021799**	RCN3	Reticulocalbin 3	1.241	0.05
**ENSBTAG00000015089**	LGALS1	Galectin-1	1.211	0.05

1 Positive log fold change indicates upregulation in abscessed (ABS) relative to healthy liver capsule samples.

**Table 2:** Downregulated differentially expressed genes within Glisson’s capsule for cattle with a liver abscess.

Name	Gene symbol	Gene name	Log2-fold change^1^	False discovery rate *P-*value
**ENSBTAG00000024957**	SNCA	Alpha-synuclein protein	−2.095	< 0.01
**ENSBTAG00000047914**	CDHR5	Cadherin-related family member 5	−2.034	< 0.01
**ENSBTAG00000018707**	ZASP	Z-band alternatively spliced PDZ-motif protein	−1.975	< 0.01
**ENSBTAG00000058262**	MAT1A	Methionine adenosyltransferase 1A	−1.421	< 0.01
**ENSBTAG00000019616**	APCS	Serum amyloid P-component	−1.631	0.01
**ENSBTAG00000078244**	—	—	−1.495	0.01
**ENSBTAG00000007616**	TMED6	Transmembrane P24 trafficking protein 6	−1.264	0.01
**ENSBTAG00000025219**	SLFN12	Schlafen family member 12	−1.056	0.01
**ENSBTAG00000072476**	NID1	Nidogen-1	−1.032	0.01
**ENSBTAG00000076719**	—	—	−2.387	0.02
**ENSBTAG00000078029**	INMT	Indolethylamine N-methyltransferase	−1.419	0.02
**ENSBTAG00000061094**	ABCC4	Multidrug resistance protein 4	−4.477	0.03
**ENSBTAG00000076667**	—	—	−1.358	0.03
**ENSBTAG00000076725**	—	—	−1.248	0.03
**ENSBTAG00000005069**	TM7SF2	Transmembrane 7 superfamily member 2	−1.053	0.03
**ENSBTAG00000062510**	BCL11A	B-cell lymphoma/leukemia 11A	−1.586	0.05
**ENSBTAG00000070571**	—	—	−1.360	0.05
**ENSBTAG00000073268**	—	—	−1.070	0.05
**ENSBTAG00000072144**	—	—	−1.030	0.05

1 Negative log2 fold change indicates downregulation in abscessed (ABS) relative to healthy liver capsule samples.

**Table 3:** Functional enrichment analysis of differentially expressed genes within Glisson’s capsule.

Source	Term name	Adjusted *P*-value	Genes overlapping (*n*)	Gene IDs
***Up regulated^1^***				
**GO: CC**	Extracellular region	0.0174	8	*IGSF10, PDGFRL, C1QTNF6, COL6A6, LOX, SPARC, LGALS1, LIPG*
**WP**	Corticotropin releasing hormone	0.0088	3	*NR4A2, FOS, FOSB*
**WP**	EGF–EGFR signaling pathway	0.0477	3	*FOS*, *FOSB*, *NR4A2*, *LGALS1*, *SPARC*, *LOX*, *P4HA2*, and *FZD2*
***Down regulated^2^***				
**GO: MF**	Calcium ion binding	0.0273	3	*APCS, SNCA, CDHR5*

1 Up-regulated genes are defined as genes with increased expression in Glisson’s capsule tissue from abscessed livers relative to Glisson’s capsule tissue from healthy livers.

2 Down-regulated genes are defined as genes with decreased expression in Glisson’s capsule tissue from abscessed livers relative to Glisson’s capsule tissue from healthy livers.

## Discussion

Glisson’s capsule is an extracellular matrix surrounding liver tissue. This connective tissue layer is primarily comprised of types I and III collagen and allows for subtle fluctuations in liver size from day to day. Previous studies on human livers have reported a dramatic three-fold increase in capsule thickness in patients with cirrhosis (Llewellyn et al. 2023). Cattle and other larger mammals have a particularly well-developed capsule (Uchida et al. 2025); Glisson’s capsule tissue in humans is 70.1 µm in thickness (Llewellyn et al. 2023), in comparison to bovine (79.1 µm), horse (125.1 µm), and swine (50.4 µm).

Prior to this study, gene expression within Glisson’s capsule had not been characterized. The gene with the largest increase in expression in capsular tissue from abscessed livers was *TLH29* protein-like *(ISG12(B))*, also known as interferon-stimulated gene 12B. The protein produced from this gene is an interferon-inducible mitochondrial protein implicated in innate immune and inflammatory signaling in mice cells (Lu and Liao 2011) and mitochondria-mediated apoptosis in bovine blastocysts (Kim et al. 2025). Upregulation of the *ISG12* family, including *IFI27*, has been observed in inflamed gastrointestinal tissues, such as colon samples from human patients with inflammatory bowel disease (Dooley et al. 2004), as well as in immune cells during acute systemic inflammatory responses such as endotoxemia and polymicrobial sepsis in mice (Uhrin et al. 2013). Elevated *ISG12B* expression may reflect heightened local inflammatory activation and tissue stress within Glisson’s capsule in livers affected by abscesses.

Another gene of interest that showed increased expression in capsule tissue from abscessed livers was receptor protein-tyrosine kinase *(EPHB3). EPHB3* encodes a receptor tyrosine kinase of the EphB subclass, which binds membrane-anchored ephrin-B ligands to mediate bidirectional cell–cell signaling that regulates axon guidance, cell migration, and tissue patterning during development (Barquilla and Pasquale 2015). EphB receptors, including EphB3, are highly expressed in the intestinal epithelium, and EphB family members are present in enteric neurons of the myenteric and submucosal plexuses (Bodin et al. 2021). EphB/ephrin B signaling contributes to intestinal epithelial wound healing and barrier maintenance, modulates enteric neuroimmune interactions, and promotes synaptic plasticity associated with visceral hypersensitivity and inflammatory abdominal pain in mice with colitis (Verma-Gandhu et al. 2007). In the setting of LA-associated inflammation, elevated *EPHB3* expression may reflect engagement of EphB/ephrin-B signaling pathways involved in inflammatory tissue remodeling and neuroimmune interactions that have been linked to peripheral pain signaling in other systems.

*c-Fos* is an immediate early gene whose expression is rapidly induced by diverse stimuli and is widely used as a marker of cellular activation, reflecting engagement of stimulus-responsive transcriptional programs in mice, rats, cats, monkeys, humans, and dogs (Aparicio et al. 2022). In pain pathways, high-threshold noxious input from peripheral tissues induces robust *c-Fos* expression in spinal dorsal horn neurons, where it serves as a well-established marker of nociceptive neuronal activation in rats and activity-dependent transcriptional responses associated with pain processing (Ahmad and Ismail 2002; Gao and Ji 2009). Increased *c-Fos* expression in the Glisson’s capsule of abscessed livers may reflect acute tissue injury, sudden pressure changes, or a fresh wave of inflammation from rapidly expanding LA formation, consistent with its broader role as an immediate-early gene responsive to diverse cellular stimuli across multiple cell types.

The *FOSB* gene had elevated expression in the abscessed capsular tissue relative to the healthy tissue. *FOS* family genes are broad immediate-early transcription factors associated with a variety of pathways and processes throughout the animal body, which can include triggering nerve cell activity (Marx 1987), cell differentiation and proliferation (Deschamps et al. 1985), and tissue homeostasis (Durchdewald et al. 2009). *FOS* and *FOSB* genes are widely associated with tumorigenic processes (Cordier and Creytens 2023), and *FOS* expression has been traced back to environmental stimuli that stimulate the adaptive response system (Brown et al. 1996). While transcriptomic data alone cannot establish a causal link to nociceptive signaling specifically, one relevant precedent comes from a study conducted by Luis-Delgado et al. (2006), which demonstrated that ΔFosB may be a marker of sustained pain when evaluating paw injury in mice. Unlike c-Fos, isoforms of *FOSB* are exceptionally stable and accumulate slowly over time in response to repeated, chronic stimulation. As with bulk RNA-seq data generally, FOSB expression here reflects the capsular tissue as a whole, encompassing neuronal, fibroblast, immune, and other resident cell populations, rather than a single, isolated cell type. Glisson’s capsule is a fibrous connective tissue layer innervated by sensory nerve fibers and populated by fibroblasts, immune cells, and vascular components, and elevated FOSB could originate from neuronal populations consistent with nociceptive activation or from fibroblast and immune populations consistent with a broader inflammatory or stress response. Elevated *FOSB* in the capsule may be consistent with chronic, long-term visceral discomfort and sustained mechanical strain that the animal may have endured.

Synuclein Alpha (SNCA) exhibited decreased expression (log2FC = −2.095) in the abscessed capsular tissue. Synuclein Alpha codes for the α-synuclein protein and is expressed within the central nervous system of animals and has been associated with a multitude of terminal neurological diseases when overexpressed, including Alzheimer’s and Parkinson’s (Cheng et al. 2011; Alecu and Bennett 2019). α-synuclein has additionally been reported to behave like prions in cases of overexpression (Melki 2018). Previous studies have demonstrated *SNCA* to become more sensitive in environments with oxidative stress (Flierl et al. 2014). The downregulation of *SNCA* expression can lead to dopamine neuron synthesis and increase the production of dopamine metabolites (Perez et al. 2002).

The gene with the largest absolute difference in expression was ENSBTAG00000061094, which is currently annotated in Ensembl as a novel bovine gene with limited functional characterization. Orthology analysis identified ATP-binding cassette subfamily C member 4 (*ABCC4*) as its human ortholog, which codes for multidrug resistance protein 4 (*MRP4*). *ABCC4* is a member of the ATP binding cassette transporter family that uses ATP hydrolysis to export a range of small endogenous and exogenous molecules, including signaling mediators such as cyclic nucleotides, urate, and inflammatory eicosanoids (for example, PGE2 and LTB4), across the plasma membrane (Russel et al. 2008). Genetic disruption of *MRP4* in mice diminishes peripheral PGE2 signaling and elevates inflammatory pain thresholds, indicating that *ABCC4* activity modulates inflammatory nociceptive responses (Lin et al. 2008). The marked downregulation of *ABCC4* in capsule tissue from abscessed livers may reflect altered local handling of inflammatory mediators, with potential consequences for nociceptive signaling and inflammation in affected tissue. Previous researchers (Felizari et al. 2025) reported *HSPA6* to be upregulated in livers with a LA.

Eight genes (Table 3) that showed increased expression were enriched for the cellular component “extracellular region.” This is biologically plausible given that Glisson’s capsule is a dense connective tissue layer formed by fibroblasts and dominated by extracellular matrix, with a particularly high abundance of fibrillar collagens, including collagens I and III (Chapman and Eagles 2007; Klaas et al. 2016). In liver disease, the capsule becomes thicker and more cellular and shows marked changes in extracellular matrix composition and organization, indicating that it is an active site of injury-related remodeling rather than a passive covering (Llewellyn et al. 2023). The enrichment of “extracellular region” reflects the involvement of structural and matrix-associated proteins broadly linked to tissue remodeling, a process consistent with the physical and inflammatory changes expected in abscessed capsular tissue.

Pathway-level analysis using WikiPathways highlighted significant enrichment of “corticotropin-releasing hormone” (*FOS*, *FOSB*, and *NR4A2*) and “EGF–EGFR signaling pathway” (*FOS*, *FOSB*, *NR4A2*, *LGALS1*, *SPARC*, *LOX*, *P4HA2*, and *FZD2*), suggesting coordinated regulation of stress-responsive and growth factor-mediated signaling in capsular tissue. Both *FOS* and *FOSB* genes appear in these pathways, an indication of both stress and inflammatory signaling.

The only significant functional enrichment result identified among the downregulated genes was the molecular function “calcium ion binding.” “Calcium ion binding” refers to the ability of proteins to reversibly bind Ca²⁺, a property common to proteins involved in structural, regulatory, or interaction-mediated cellular processes across many cell types and biological functions. Calcium-dependent signaling has been implicated in nociception by regulating cellular excitability, neurotransmitter release, and transmission of nociceptive signals (Zhang et al. 2025), however, calcium ion binding is a broad functional category involved in numerous non-nociceptive cellular processes as well. The reduced expression of genes associated with calcium ion binding suggests altered calcium-dependent cellular activity in Glisson’s capsule surrounding abscessed livers. Calcium signaling is a broad and fundamental process across many cell types, and this finding is consistent with the sensitized cellular environment described in the abscessed capsule, adding to the broader pattern of evidence suggesting altered cellular activity in response to abscess presence.

Without cellular localization or protein-level data, transcriptomic evidence alone cannot definitively distinguish nociceptive signaling from general inflammation, or separate cell-intrinsic expression changes from shifts in cellular composition. Further research with larger sample sizes should confirm these associations through histological and immunohistochemical assessment of aspects such as nerve fiber density, protein-level investigation, and RT-qPCR validation of key genes (FOS, FOSB, EPHB3, etc.), to clarify the animal welfare implications of bovine LA.

To our knowledge, this is the first study to compare gene expression in Glisson’s capsule between livers with and without abscesses, offering an initial molecular perspective on noxious stimuli associated with this condition. Given the modest sample size and lack of covariate adjustment, this differential expression results represent a novel exploratory look at capsular transcriptomic changes associated with LA. The 60 genes identified were consistently associated with inflammation pathways, nociceptive signaling, and tissue stress, providing early molecular evidence that the liver capsule from abscessed livers may exist in a primed, hyper-sensitized neuro-inflammatory state rather than experiencing passive mechanical stretching alone.

## Data Availability

The data underlying this article will be shared on reasonable request to the corresponding author.

## Abbreviations

LA: liver abscess
RIN: RNA integrity numbers
TIN: transcript integrity numbers
ABS: abscessed liver
